# Exploration of truss metamaterials with graph based generative modeling

**DOI:** 10.1038/s41467-023-43217-y

**Published:** 2023-11-21

**Authors:** Angkur Jyoti Dipanka Shaikeea

**Affiliations:** Department of Engineering, University of Cambridge, Cambridge, UK

**Keywords:** Mechanical engineering, Mechanical properties

## Abstract

Optimisation tasks in the inverse design of metamaterials with machine learning were limited due to the representations of generative models. Here the author comments a recent publication in Nature Communications which generates a latent space representation that unlocks non-linear optimisations.

## Teaser

In the expanding landscape of metamaterial design, Zheng and colleagues introduces a framework that bridges design and properties, using machine learning to enhance truss metamaterials. A neural network creates an interpretable, low-dimensional space, empowering designers to tailor mechanical properties.

The ever expanding landscape of metamaterial design has experienced a significant breakthrough thanks to the emergence and continuous development of additive manufacturing, allowing for the creation of cellular solids with intricately tailored microstructural architectures^1^. This breakthrough has unlocked a realm of extraordinary functionalities that include counterintuitive negative compressibility^2^, negative Poisson’s ratios^3^, mechanical cloaking^4^, extreme energy absorption^5^, and guided acoustic waves^6^. Amidst this array of design possibilities, truss metamaterials, characterized by periodic lattices of beam networks, have emerged as central players. Their appeal lies in their ultralow relative-density strength and ease of manufacturability, yet their full potential remains largely untapped.

Truss metamaterials offer an enticing landscape for innovation with their versatile design space based on lattice topology and geometric attributes. However, this richness has often been limited to a handful of ad-hoc lattices discovered through intuition and trial-and-error. The quest for optimal truss designs faces hurdles due to the computational complexity of exploring the vast and noisy search space. Traditional solutions, including heuristic search strategies, struggle to scale. Even recent efforts to categorize truss lattices akin to molecular structures have grappled with the challenge of discontinuous design spaces^7^.

## Metamaterials as graphs with a creative touch

Recent research has harnessed graph representations to interpret trusses as data structures resembling 3D nodal coordinates and connectivity information, akin to molecules^8, 9^. While most prior work has translated lattices into graphs and tackled the forward problem of predicting property-structure relationships, a recent article titled “Unifying the design space of truss metamaterials by generative modelling” by Zheng^10^ and colleagues introduces a very creative machine learning (ML) framework. This framework constructs a low-dimensional, continuous latent representation of graph data, effectively bringing together an extensive range of metamaterial designs. This achievement is realized through a neural network architecture, specifically a variational autoencoder, which creates an informational bottleneck, enabling compression of the high-dimensional graph representation into a finite, low-dimensional, and smooth vector representation. This latent space facilitates the generation of lattice designs through random sampling, encapsulating truss connectivity, node positions, and shared information in distinct dimensions, thereby providing comprehensible insight into a previously enigmatic design space.

Historically, the exploration of structure-property relationships in metamaterials has followed a forward trajectory, which is, given a design, effective properties are derived through computational or experimental characterization. In contrast, the inverse challenge—identifying a material design that meets specific mechanical property requirements—has often relied on inefficient trial and error or researchers’ intuition. This has underscored the need for ML methods to expedite the design process and efficiently uncover metamaterials with tailored properties.

## Accelerating the inverse design of metamaterials

The challenge of inverse design is particularly compounded by the absence of a standardized design parameterization. Most truss lattices are based on recognizable topologies such as the diamond, Kagome, octahedron, or honeycomb lattices. However, these topologies are labelled in an ad-hoc manner, lacking consistent and rigorous design conventions that can be applied uniformly across different lattice types. Each of these topologies can be defined using a manually crafted finite set of design parameters, such as strut lengths and orientations. Regrettably, these parameters cannot be generalized effectively to encompass other lattice topologies. For instance, while the distinction between the Kagome and octahedron topologies is clear to human observers, it does not possess direct cognitive relevance to a computer algorithm striving to optimize the design. As a result, most existing design optimization methods for truss lattices have concentrated solely on a limited selection of topologies. This approach yields suboptimal solutions and neglects the full spectrum of potential lattice structures available. Hence, the pressing need for a method that can harmonize the design space of truss metamaterials.

Towards this end, Zheng and colleagues reduced the discrete design space of lattice parameters to continuous space of latent variables of the autoencoder. This technique, which is central in nonlinear model order reduction methods, is now utilized to distil the underlying manifold of the lattice structures and their mechanical behaviour. However, the latent space suffers from the issue that the reduced variables are not interpretable in general, which leads to a challenge to understand the relationship between latent spaces with physical attributes. The creativity of the authors lies in their ability to decompose the latent space in physically distinct topology-specific, geometry-specific and their shared dimensions. While topology-specific dimensions embed information about connectivity of truss structures, geometry-specific dimensions characterize strut length, diameter. The ability to independently change topology or geometry introduces a modularity in design that is absent in existing optimization techniques. The practical implication of the decomposition is significant as well since it can empower designers to understand the design process and make an informed decision to cater to specific design goals. Beyond human interpretability, the decomposition also improves ability of the framework to generalize to domains absent in training samples.

## Optimization with machine learning and gradient methods

The integration of the ML framework with gradient-based optimization has yielded fruitful results. The framework has been used to design graph-based truss lattices for metamaterials, tailoring mechanical properties across linear and notably nonlinear regimes. This includes designs with exceptional stiffness, auxetic behaviour, and tailored stress–strain responses. The interpretable latent space allows separate or combined adjustments to topology and geometry, offering designers a remarkable degree of control. The unified latent space extrapolates creatively, enabling the discovery of lattice designs far beyond the known design and data space, achieving stiffness, auxetic behaviour, and unique stress-strain characteristics.

The framework proposed by Zheng and team stands out in its ability to spark creativity. Analogous to an artist’s palette, the latent space empowers effortless generation of truss structures through elegant operations—sampling around familiar data points, traversing latent axes, and seamlessly interpolating between two points. In contrast to conventional methods that often obscure physical insights, the neural network architecture of this framework bridges the gap by predicting truss properties from the latent space. This fusion of generative modelling and predictive analysis equips researchers to explore uncharted territories and unveil designs that defy convention.

**Fig. 1:**
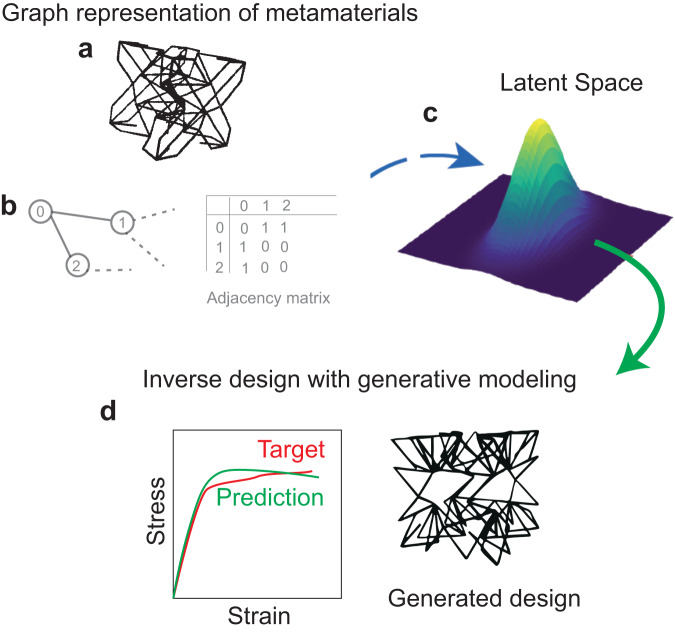
Graph based generative modelling of metamaterials. **a** Complex truss lattices are represented as **b** graphs with appropriate nodal properties and connectivity, encoded in an adjacency matrix. **c** To avoid optimization over discrete space of lattice parameters, such as strut length, diameter, the lattice structures are represented in a latent space with appropriate decompositions in physically distinct dimensions. This enables human interpretability and generalizability of the neural network, thus addressing **d** the inverse problem of generating truss topologies from target properties, both in linear and nonlinear response.

Zheng and colleagues’ framework has combined the topological characterization and mechanical response in a modular fashion, which enables them to obtain optimized lattice structures for linear or nonlinear stress-strain behaviour in rather ease (Fig. 1). Extension to nonlinear behaviour with complicated topology of lattices could lead to complicated behaviour. This modular character of the framework allows them to obtain lattices that have been optimized for multiple properties simultaneously. This can be achieved by just multitask property predictor, while leveraging correlations and shared topological information of the underlying lattices. Thus, the framework could open a new direction of the field by significantly speeding up the optimization process for lattice structures that need to meet complex design requirements. For example, if we can design lattice structures that are simultaneously optimized for strength, toughness, it could output structural components in aerospace, civil engineering and even biomedicine.

## Future directions

Numerous unanswered questions and unexplored territories persist in the realm of metamaterial design. Firstly, the acquisition of experimental data remains imperative for comprehending how the simulated behaviour of intricate metamaterials aligns with their actual performance. Additionally, the incorporation of imperfections in additively printed metamaterials into a machine learning framework can shed light on their impact on mechanical performance^11^. In the recent work by Zheng and colleagues, the scope of considered topologies is confined to cubic symmetry. However, there exists room to broaden this scope, encompassing highly anisotropic lattice designs. Furthermore, the material properties need not adhere to linearity; instead, they can span a spectrum from hyperplastic polymers to metal plasticity^12^, thereby introducing another layer of non-linearity into the design process.
